# Insulin therapy for type 2 diabetes – are we there yet? The d-Nav® story

**DOI:** 10.1186/s40842-018-0056-5

**Published:** 2018-04-10

**Authors:** I. Hodish

**Affiliations:** 1 Department of Internal Medicine, Division of Metabolism, Endocrinology and Diabetes, University of Michigan Medical Center, 1000 Wall St, Ann Arbor, MI 48105 USA; 2 Hygieia, Inc, Livonia, MI USA

**Keywords:** Insulin therapy, Hypoglycemia, Dosage, Type 2 diabetes

## Abstract

Insulin replacement therapy is mostly used by patients with type 2 diabetes who become insulin deficient and have failed other therapeutic options. They comprise about a quarter of those with diabetes, endures the majority of the complications and consumes the majority of the resources. Adequate insulin replacement therapy can prevent complications and reduce expenses, as long as therapy goals are achieved and maintained. Sadly, these therapy goals are seldom achieved and outcomes have not improved for decades despite advances in pharmacotherapy and technology.

There is a growing recognition that the low success rate of insulin therapy results from intra-individual and inter-individual variations in insulin requirements. Total insulin requirements per day vary considerably between patients and constantly change without achieving a steady state. Thus, the key element in effective insulin therapy is unremitting and frequent dosage adjustments that can overcome those dynamics. In practice, insulin adjustments are done sporadically during outpatient clinic. Due to time constraints, providers are not able to deliver appropriate insulin dosage optimization.

The d-Nav® Insulin Guidance Service has been developed to provide appropriate insulinization in insulin users without increasing the burden on healthcare systems. It relies on dedicated clinicians and a spectrum of technological solutions. Patients are provided with a handheld device called d-Nav® which advises them what dose of insulin to administer during each injection and automatically adjust insulin dosage when needed. The d-Nav care specialists periodically follow-up with users through telephone calls and in-person consultations to bestow user confidence, correct usage errors, triage, and identify uncharacteristic clinical courses.

The following review provide details about the service and its clinical outcomes.

## Background

### Index cases

**Fig. 1:**
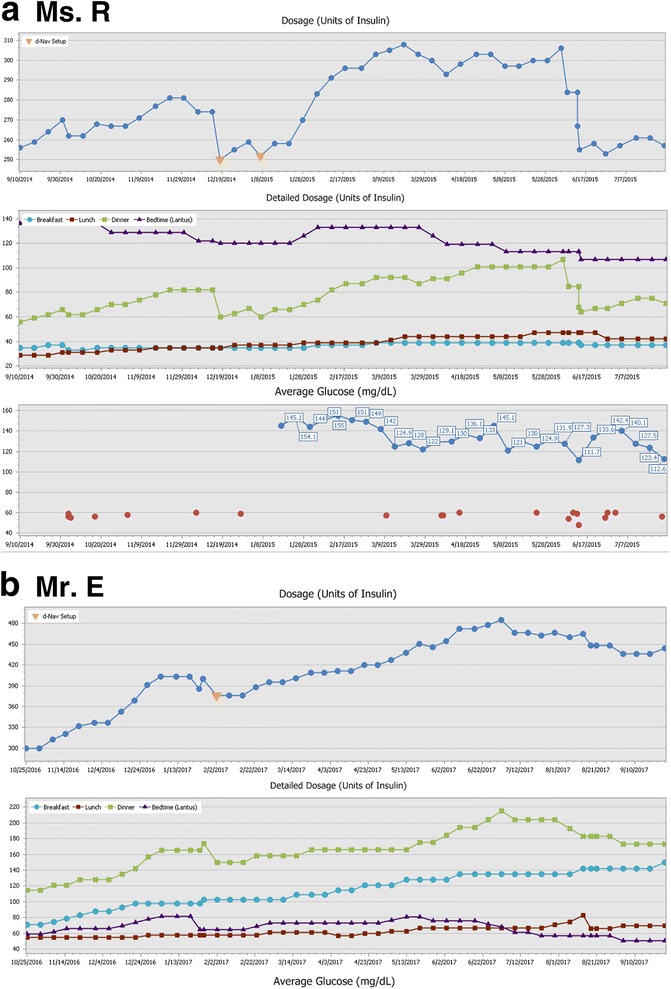
d-Nav downloads for the index patients. The upper graph denotes total daily insulin over time. The lower graph denotes each component of the patient dosage, including long-acting insulin analog before bed and rapid-acting insulin analog with breakfast, lunch and dinner. Correction factors are not shown. **a**) Ms. R.; **b**) Mr. E

Ms. R. is a 69-year-old woman with type 2 diabetes who has been treated with insulin since 2011. She is currently treated with basal-bolus insulin therapy that consists of once daily long-acting insulin analog and 3 rapid-acting insulin analog boluses with meals. Her diabetes control was poor for years, and in 4/2013 her A1c was 9.8%. Since 7/2014, her glycemic control has improved considerably with A1c levels ranging between 6.9% and 7.7%. She typically measures glucose 4 times/day before all meals and before bedtime. The frequency of her recorded hypoglycemia (glucose < 60 mg/dl) has been low at about 0.5 events per week without any nocturnal events. To achieve good glycemic control on insulin therapy, her insulin dosage has been adjusted 48 times over the past 28 weeks (see details in Fig. 1a). Notably, her total daily insulin was increased by about 15% for a period of 5 months before it decreased to the original daily insulin dosage. Most of her dosage changes occurred in her long-acting insulin dose and her dinner rapid-acting insulin dose, although other components have changed as well.

Mr. E. is a 61-year-old man with type 2 diabetes who has been treated with insulin for 20 years. He is currently treated with basal-bolus insulin therapy. His diabetes control was poor for years and in 8/2015 his A1c was 9.1%. Since 11/2015, his glycemic control has improved considerably with A1c levels ranging between 5.5% to 6.6%. Lately, he has been measuring glucose about 23 times per week, regularly skipping pre-dinner tests. The frequency of his recorded hypoglycemia (glucose < 60 mg/dl) has been low at 0.2 events per week with a single nocturnal event for the past year. To achieve good glycemic control on insulin therapy, his insulin dosage has been adjusted 49 times over the past 32 weeks (see details in Fig. 1b). Between the beginning of 2017 and 6/2017, his insulin requirements and hence total daily insulin gradually increased by about 60% until it steadied in the beginning of 6/2017. Despite this relative stability in total daily insulin requirement, each component of his therapy continued to change. For example, dinner rapid-acting insulin bolus decreased by about 20%, breakfast increased by about 7% and long-acting insulin decreased by about 35%.

**Fig. 2:**
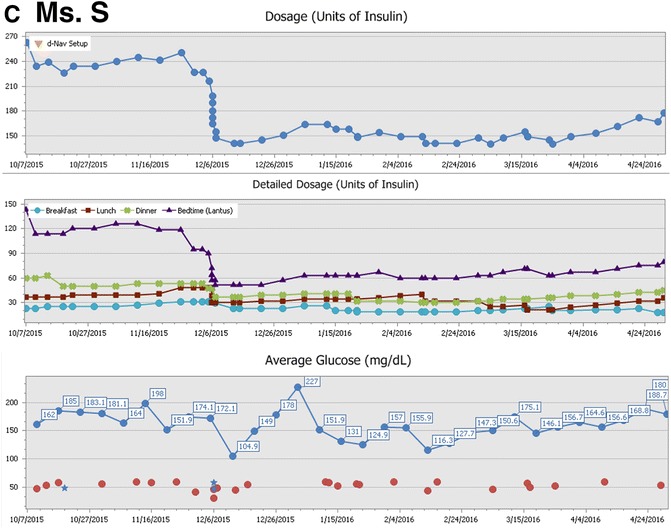
The graph denotes weekly mean glucose (in mg/dl). Episodes of minor daytime hypoglycemia are shown in the lower graph as red dots (glucose < 60 mg/dl). Nocturnal events are shown as blue stars. **c**) Ms. S

Ms. S. is a 62-year-old woman with type 2 diabetes who has been treated with insulin for 15 years. She is currently treated with basal-bolus insulin therapy. Her diabetes control was inadequate for years and in 2/2013 her A1c was 11.7%. Since 7/2013, her glycemic control has improved considerably with A1c levels ranging between 6.8% to 7.6%. Lately, she has been measuring glucose about 20 times per week, missing about a third of the times before lunch and bedtime. In 11/2015, her insulin requirements decreased by about 40% over a period of 2 weeks without warning or clear clinical reason. This reduction occurred mainly in her long-acting insulin dosage component, and to some extent in her lunch and breakfast boluses. After this noteworthy dosage reduction occurred, the frequency of her recorded hypoglycemia (glucose < 60 mg/dl) has been low at 0.7 events per week without any nocturnal events. To achieve good glycemic control on insulin therapy, her insulin dosage has been adjusted 50 times over the past 30 weeks (see details in Fig. 2c).

The above examples represent 3 typical patients with type 2 diabetes who use insulin. They have required considerable clinical effort in insulin titrations to improve and maintain appropriate diabetes control. In reality, most specialized providers are expected to provide care for hundreds of patients using insulin. Is it realistic to expect healthcare professionals to provide such a level of care to their patients?

In fact, the 3 examples are patients whose insulin therapy has been managed by the d-Nav® Insulin Guidance Service. The providers who referred these patients to the service had the ability to supervise the process but were not involved in the process of insulin dosage titration.

### The “insulin paradox”

Patients with hormonal deficiencies are commonly seen in endocrinology practices. The endocrinologist is expected not only to diagnose the deficiency but also to fully replace it with adequate dosage of the appropriate hormone. Fundamentally, Inappropriate hormonal replacements such as hypothyroidism, adrenal insufficiency, or hypogonadism are considered as clinically unacceptable and poor quality-markers for the clinician. In contrast, replacement of the most common hormone deficiency, namely insulin deficiency (i.e., diabetes), is ineffective in the majority of the cases, even in the most reputable clinics and predominantly blamed on the patient.

Insulin replacement therapy is mostly used by patients with type 2 diabetes who become insulin deficient and have failed other therapeutic options (herein referred to as advanced diabetes). During the first decade with the disease, oral or non-insulin injectable medications that enhance endogenous insulin secretion and alleviate its resistance [1–4], are mostly suitable and effective in maintaining adequate glycemia. The clinical picture changes typically toward the second decade, during which many patients progress their insulin deficiency to the extent that insulin replacement therapy is warranted [5]. Initially, many patients can do well with simple regimens such as long-acting insulin only, yet over a period of a few years most require some level of fast acting insulin coverage to maintain appropriate glycemia [6]. About a quarter of the patients with type 2 diabetes use insulin. This cohort endures the majority of the complications and consumes the majority of the resources [7].

Although insulin as a hormone possesses multiple metabolic functions [8], the main therapeutic marker used to assess therapy adequacy is average glucose, or glycated hemoglobin (A1c). Studies have shown that adequate insulin replacement therapy prevents complications and premature death as long as therapy goals are achieved and maintained [9, 10]. Undoubtedly, supervision of statins and maintenance of normal blood pressure are vital [11]. The importance in reassuring appropriate insulin availability transpires beyond preventing glycemic damage [12]. Emerging data has shown that loss of insulin signaling in critical organs, worsens atherosclerosis and nephropathy in normoglycemic animals [13–15].

Most authorities recommend an A1c goal between 6.5% (and even lower if feasible) to 7.5% for the majority of patients [16–18]. Sadly, these therapy goals are seldom achieved and outcomes have not improved for decades despite advances in pharmacotherapy and technology. The average A1c in insulin users in the USA (as in Europe) is approximately 8.5% and a third of users continue to experience A1c at 9% or higher [19–21].

This phenomenon (also called the “insulin paradox”) is peculiar given the benefit of the drug and its safety profile. Insulin has been available for almost a century and exists in a variety of formulations with different pharmacokinetic and pharmacodynamic profiles. Compared to all other diabetes drugs, insulin does not have an upper dosage limit, and therefore, there is no level of hyperglycemia it cannot overcome. The only considerable side effect related to insulin therapy is hypoglycemia. It is the only known side effect that has been shown to be directly related to insulin and may be hazardous [22, 23]. Hypoglycemia has been experienced by most patients with insulin and it is the main limiting factor for effective insulin therapy. Severe hypoglycemia is the main aspect of hypoglycemia that has been feared by patients and clinicians alike. Tremendous amount of research has been made to understand and prevent these severe events [24, 25]. Intriguingly, in prospective clinical studies, for each individual who dies of severe hypoglycemia, hundreds die from other conditions of which many are related to diabetes and may potentially be preventable [26, 27].

Administration of insulin is largely painless, and the level of patient compliance is not different from compliance with other medications used to treat diabetes or other medical conditions such as hypertension [28–30]. It has been shown that about a quarter of patients with chronic medical conditions (including diabetes) do not follow providers’ recommendations [28]. Therefore, limited compliance doesn’t explain why most patients using insulin do not achieve their therapy goals.

There is a growing recognition that the “insulin paradox” results from intra-individual and inter-individual variations in insulin requirements. Normal pancreases secrete about 1 unit of insulin per kg body weight per day [31–34]. Endogenous pancreatic insulin secretion occurs in the portal system; whereas, the main organ responsive to the hormone is the liver. Under physiological settings, the liver retains about 85% of the digested glucose [31]. Once a patient loses their ability to secret enough insulin and requires insulin replacement therapy, the insulin is administered peripherally, outside the portal system. Due to peripheral metabolism of the hormone, mainly in the kidney, the required dosage needed to achieve similar levels of insulin in the portal system doubles [35]. This does not take into account cutaneous degradation of injected insulin that has been found to be considerable in some patients [36]. Endogenous insulin resources likely diminish gradually, as it may take a few years to build the required individual daily dosage once insulin therapy is initiated [6]. Although patients gain about 5 kg with insulin initiation, this weight gain plateaus within a year and therefore does not explain the gradual increase in insulin requirements that can last as long as 3 years [6]. In the majority of cases patients’ fluctuations in blood glucose and their tendency to develop hypoglycemia worsens after a few years [37]. Not surprisingly, in clinical studies that supervise treat-to-target insulin therapy in patients with type 2 diabetes, individual daily requirements average at 1.5–2 units per kilogram with a wide variance of distribution [38, 39]. Some patients use more than 500 units per day and occasionally utilize concentrated insulin formulations [40]. Importantly, higher dosage of insulin per day has not been shown to be associated with increased risk of hypoglycemia [41].

**Fig. 3:**
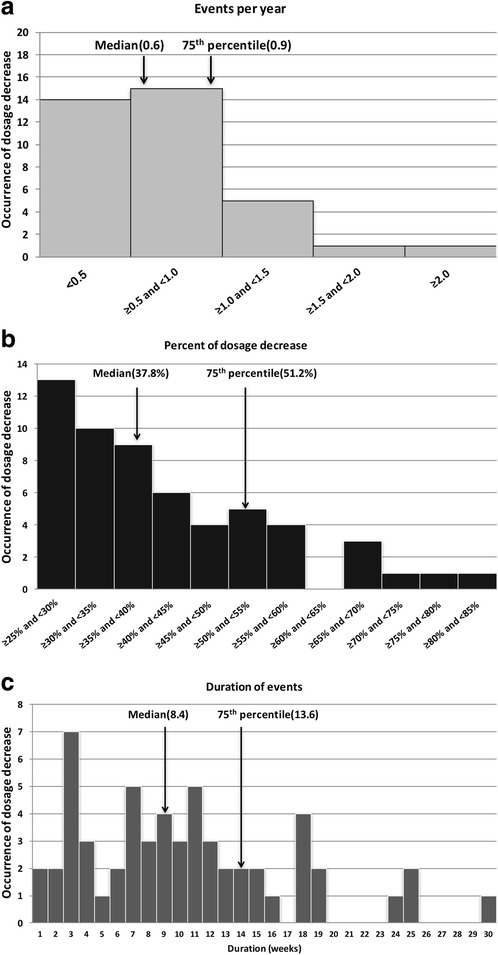
Decline in insulin requirements over time (≥25% of prior dosage) in patients with type 2 diabetes using the d-Nav Insulin Guidance Service. **a**) Histogram depicting frequency of dosage reduction per year. In half of the cases, events occurred more than 0.6 times per year. **b**) Histogram depicting percentage of dosage reduction. In half of the cases, total daily insulin dosage decreased by more than 37.8%. **c**) Histogram depicting duration of decrease in insulin needs. In half of the cases, duration of the period exceeded 8.4 weeks. The graphs were reproduced from an already published information; Harper et al. [52]

Insulin needs do not reach a steady state in patients with either type 1 or type 2 diabetes, as shown in the index cases in this review (Figs. 1 and 2). The need to constantly titrate insulin dosage in order to maintain euglycemia was demonstrated in clinical studies which supervised frequent insulin titrations both in the context of type 1 [42, 43] and type 2 diabetes [39, 44–51]. For most patients, insulin needs are unpredictable and dynamic, with requirements changing by 40% or even more (Fig. 3) [52, 54]. Unpredicted changes in insulin requirements expose patients to hypoglycemia or hyperglycemia [52–54].

Consequently, the key element in effective insulin therapy is unremitting and frequent dosage adjustments that can overcome those dynamics and enable maintenance of optimal glycemic control while minimizing occurrences of hypoglycemia [50, 53–56]. Judging from commonly used titration protocols, weekly titrations are preferable [39, 50, 51]. Simply stated, insulin is likely one the most dynamic drug existing in modern medicine. Not surprisingly, A1c goals have been achieved and maintained primarily in clinical trials that implement insulin dosage adjustment every few days-weeks [39, 44–51]. This beneficial effect lasts only as long as periodic adjustments are made by the medical staff, evidenced by deterioration of glycemic control within a few months after the studies end and insulin titrations became more sporadic [57, 58]. In practice, insulin adjustments are done sporadically during outpatient clinic visits every 3–6 months, which explains why the effectiveness of one of the earliest and most promising drugs in modern medicine has largely been poor. Patients are not typically blamed for inadequate blood pressure management or persistent elevated LDL, nor should they be blamed for inadequate insulin replacement therapy.

Insulin dosage titration is a teachable skill, yet it is time consuming and requires resources that are only available in well-funded trials. For illustration, it may take more than 15 min to contact a patient, deliberate, and convey adjustment in dosage. Even if a provider did nothing else but adjust weekly insulin dosage, he/she would only be able to support about 150 patients each. In the USA, there are about 7,000,000 patients who use insulin (www.cdc.gov/diabetes/statistics/meduse/fig2.htm). Thus, over 44,000 providers dedicated only to insulin dosage adjustments would be required to effectively support patients via means such as telemedicine. Currently, in the USA, there are about 15,000 providers who possess the required expertise [59] (https://www.diabeteseducator.org/about-aade), yet their time is already fully committed. Even every-other-week insulin dosage adjustments would overwhelm the healthcare system. Most importantly, the challenge is not in the collection and delivery of glucose data to the provider, for which a breadth of advanced technological solutions is available. It is not even in the deliberation process and medical decision-making. The main challenge is the need to close the loop; to deliver the recommendation to the patient in a way that he/she understands and can comfortably incorporate into daily life until the next adjustment is needed.

The prevalence of type 2 diabetes is on the rise; however, the availability of providers with expertise in insulin therapy is globally stable and low. As mentioned above, due to time constraints, providers are not able to deliver appropriate insulin dosage optimization. Accordingly, the only solution to the problem is one that provides frequent insulin dosage titrations in a way that the success of the therapy does not increase the burden on health care systems.

### The d-Nav® insulin guidance service

Hygieia, Inc. was spun out of the University of Michigan and founded in 2008 by Israel Hodish, MD, PhD and Eran Bashan, PhD. The company has developed a scalable solution to the problem in the form of an integrated service that provides appropriate insulinization in insulin users. The service relies on dedicated clinicians (also called d-Nav Care Specialists) who utilize a spectrum of proprietary technological solutions. The integrated care service model follows existing models such as: dialysis services (e.g., www.davita.com), imaging services (e.g., www.flatworldsolutions.com), home infusion (e.g., www.infusionoptions.net/index.html), physical therapy, etc.

Patients referred to the service, are provided with a handheld device called d-Nav (stands for diabetes navigator) which advises them what dose of insulin to administer during each injection. This simple to use device is CE-marked and used by patients to monitor their glucose level before each injection (not yet approved by the FDA). In turn, it provides a recommended insulin dose. Following the logic of diabetes specialists and concordant with the gold standard guidelines for insulin management [60], the device assesses the patient’s response to its current insulin dosage by analyzing glucose patterns on a weekly basis (glucose readings from the on board sensor are stored in the device), then automatically adjusts the user’s insulin dosage [53, 54, 61, 62]. The device does not require any behavioral changes from the user. Adjustments are typically made weekly. Yet, if insulin requirements drop or hypoglycemia ensues, d-Nav makes immediate adjustments as often as needed, following the safety-first approach. This dynamic insulin therapy, first closes the gap between the initial prescribed total daily dose and the therapeutic one and then constantly evaluates each component of the therapy to fit the patient’s changing needs while preventing an increase in hypoglycemia. Since d-Nav provides insulin dose recommendations it is typically used before every insulin injection, i.e., from one to four times a day depending on the regimen. d-Nav adjusts most types of insulin regimens: A) once a day basal insulin; B) twice daily biphasic/premixed long- and short-acting insulin; and, C) intensive insulin therapy involving long-acting and fast-acting insulin with or without carbohydrate counting [62].

The d-Nav care specialists periodically follow-up with users through telephone calls and in-person consultations to bestow user confidence, correct usage errors, triage, and identify uncharacteristic clinical courses (example in Fig. 2c). The d-Nav care specialist team uses software solutions that analyze a device’s data to identify usage errors and recognize atypical clinical courses. For example, the software tools help to recognize if a patient requires a different insulin regimen, such as biphasic insulin therapy instead of basal insulin only [53, 63]. These software tools are available for providers during and between clinic visits. The service is linked to a wider healthcare system such that a patient’s data is always available to be reviewed by the patient’s physicians.

### Clinical outcomes and safety

**Fig. 4:**
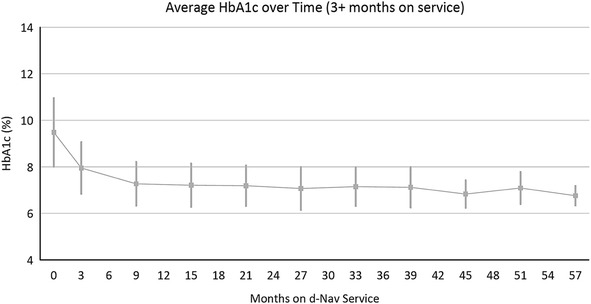
Average A1c in d-Nav users in Europe who have been in the service for more the 3 months

During the time this review was written, 1253 patients have been referred to the d-Nav service for an accumulated period of 1847 patient years. Most patients were referred with type 2 diabetes. Among active users, 339 have used the d-Nav service for more than 2 years. To date, patients’ average (±standard deviation) age is 60.8 ± 10.3 years, average duration of diabetes 15.8 ± 7.9 years and average duration of insulin usage 9.4 ± 7.5 years. Reduction in A1c is observed within 3 months of patients’ starting the d-Nav service. Most patients achieved and maintained glycemic goals for as long as they stayed with the service (Fig. 4). About 20% of the patients withdraw during the first year and less than 10% per year withdraw thereafter.

During the time this review was written, the frequency of severe hypoglycemia has been 2.2 per 100 patient years (in a cohort including patients with both type 2 and type 1 diabetes). For comparison, the expected frequency of severe hypoglycemia in insulin users with type 2 diabetes is 4–5 events per 100 patient years [25, 64]. In patients with type 1 diabetes, the frequency is higher [37].

### Cost saving

Diabetes is one of the priciest conditions to manage and its demands are on the rise. As of 2012, the estimated US expenditure for diabetes care was $245 Billion [65]. Patients with advanced diabetes, who are more likely to require insulin therapy, consume the majority of the resources.

We believe that cost saving can be realized in 3 different areas: reductions in complications and hospitalizations; reductions in outpatient clinic costs; and, reductions in pharmaceutical expenses. While the first 2 categories may require a few years of improved patients’ glycemia in order to show cost savings, the last category may exert its savings within a few months.

It has been estimated that improved glycemic control can reduce cost, mainly due to reduction in hospital admissions [66] and enhancement in the healing of foot ulcers [67, 68]. The latter has been estimated to be prevalent in up to 25% of patients with diabetes [69]. A health-economic model evaluating the cost effectiveness of the d-Nav insulin guidance service in the United Kingdom projected savings of £1459 per patient over a 3-year period in all patients, and £4992 per patient over a 3-year period in high risk patients [68].

It has been suggested that about half of the insulin treated patients with type 2 diabetes tend to have monthly physician visits [70], likely due to high frequency of complications [67]. This can potentially be alleviated within the first few years of glycemic improvement.

Most authorities consider effective insulin therapy alongside metformin to be an adequate antidiabetic regimen [17, 60]. Yet, since most patients using insulin fail to achieve adequate glycemia, other anti-hyperglycemics are typically co-prescribed, some of which are branded and expensive. This polypharmacy increases costs and the risk for side effects, and may not achieve therapy goals [71, 72]. Although some prognostic benefit has been implicated with non-insulin anti-hyperglycemics [73–76], given the prognostic implications of successful insulin therapy [9], it is not entirely clear that other pharmacotherapy is needed if insulin therapy is effective. In a health economic evaluation, 217 patients were enrolled in to the d-Nav Insulin Guidance Service through a participating insurance group. In the 192 patients who completed the first 90 days of follow up, projected direct savings from medication elimination was estimated at $1736 per patient per year for all patients, and a projected saving of $6172 per patient per year for patient who used branded medications [77].

### Competing solutions

The d-Nav Insulin Guidance Service provides insulin management, yet it by no means competes with primary care or endocrine services. Those are absolutely critical for the holistic management of the patients who often experience comorbidities and complications. There are currently 4 categories of alternative approaches for the purpose of insulin therapy optimization: data delivery platforms, insulin dose calculators, decision support systems and closed loop insulin delivery devices.

#### Data delivery platforms

As digital communication technology is advancing; a multitude of data delivery systems are becoming available. They enable real-time delivery of glucose readings from patients’ glucose monitoring devices to their physicians (e.g., http://telcare.com, https://www.livongo.com, Accu-Check® [78]). With telemedicine, physicians and their staff can talk to patients face-to-face (e.g., https://evisit.com) to provide as many medications adjustments as they see fit. However, the rate-limiting factor is the large number of patients and the frequency of dosage adjustments that they need. Therefore, data delivery platforms are unlikely to provide a solution for insulin therapy.

#### Dose calculators

A breadth of titration guidelines has been used by studies who supervised insulin therapy and some were compared [39, 79]. The similarity in success rates and the minor differences between them [49], implies that a key element in their respective success is likely related to the frequency in which they were applied. Many of the titration guidelines have been transformed into commercially available tools or apps of which some are FDA approved (e.g., https://isageapp.com, http://voluntis.com, My Dose Coach from Sanofi-Aventis). Apps can be used by patients and can enable patients to titrate their own insulin dosage with their phone. However, these apps currently require the prescribing provider to select or design an appropriate titration algorithm for each patient and to assume full responsibility for the process of dose titration. Accordingly, they still significantly increase the burden of prescribing providers.

#### Decision support systems

The process of insulin dosage titration is better managed if expertise is available. On the other hand, the number of providers who are versed in managing insulin therapy is limited and not expected to grow significantly. Existing solutions including decision support for insulin titration that can be accessed by providers (e.g., www.insulinalgorithms.com, http://www.glytecsystems.com). However, such solutions still leave the burden of conveying the new dosage to the patients on the providers side. As mentioned above, we believe that any solution that increase providers’ workload is unlikely to be successful due to the limited available time of healthcare providers.

#### Closed loop insulin delivery devices or artificial pancreas

A great deal of research have been invested to develop a closed loop system combing an insulin pump and a continuous glucose monitor to form an “artificial pancreas [80].” These systems can continuously adjust ongoing insulin delivery based on prevailing glucose (www.medtronicdiabetes.com). The main impediments to this approach is complexity. Although insulin pumps were first available in the 1980s, they are currently used by about 50% of individuals with type 1 diabetes in the US [81] and by only about 1 million users globally [82]. Continuous glucose monitor devices (CGM) were introduced over a decade ago and are currently used by a few hundred-thousand patients (e.g., Dexcom about 200,000 globally in 2016, http://investor.shareholder.com/dexcom/releasedetail.cfm?ReleaseID=1014972).

Currently, the main users of these systems are patients with type 1 diabetes. This chronic condition is hard on patients and providers alike, but relatively uncommon. Among patients with type 1 diabetes, many have stopped using either devices due to their complexity and intense requirements. It is not clear that the 7,000,000 insulin users with type 2 diabetes in the US, who are generally older than the average insulin user with type 1 diabetes, would be willing to adopt the complexity of using an insulin pump and a CGM.

#### Proof of concept

Whatever solution used to improve the care of patients using insulin, enhanced clinical outcomes and reduced cost can only be realized if clinical goals are actually achieved. Hence, proof of concept is critical. It should demonstrate not only efficacy and safety in short-term clinical trials, but also durable effect in real-life over a long period of time.

## Conclusion

Despite insulin’s long-term availability and still disappointing outcomes, we believe that achieving glycemic goals in insulin users with type 2 diabetes is within reach. When provided with frequent titration to optimize insulin dosage, the majority of patients using insulin could be maintained in a good quality of life for a longer time, while saving health care system resources.

## Abbreviations

A1c: glycated hemoglobin
CGM: Continuous glucose monitor devices
